# Research on the circular RNA bioinformatics in patients with acute myocardial infarction

**DOI:** 10.1002/jcla.23621

**Published:** 2020-10-15

**Authors:** Lianli Yin, Yinghua Tang, Minghe Jiang

**Affiliations:** ^1^ Department of Clinical Laboratory Nanning Second People's Hospital the Third Affiliated Hospital of Guangxi Medical University Nanning Guangxi China; ^2^ Department of Clinical Laboratory Guangxi Hospital Of Traditional Chinese Medicine The First Affiliated Hospital of Guangxi University of Chinese Medicine Nanning Guangxi China; ^3^ Emergency cardiothoracic Department Nanning Second People's Hospital the Third Affiliated Hospital of Guangxi Medical University, Nanning Guangxi China

**Keywords:** acute myocardial infarction, bioinformatics, circRNA

## Abstract

**Objective:**

Through the detection of circular RNA (circRNA) using expression profiling chips, we searched for circRNAs related to acute myocardial infarction (AMI) and explored their relationship and possible mechanisms with AMI.

**Method:**

The study subjects included 3 AMI patients and 3 controls, and circRNA expression profiling analysis was performed using a microarray gene chip to identify circRNAs with large differences in expression between groups and to construct a circRNA‐miRNA network.

**Results:**

Compared with the control group, there were 650 differentially expressed circRNAs found in AMI patients (*P* < .05, fold change > 2), including 535 up‐regulated circRNAs, such as hsa_circ_0050908, hsa_circRNA4010‐22, hsa_circ_0081241, hsa_circ_0010551, hsa_circRNA4010‐20, hsa_circRNA14702, hsa_circ_0115392, has_circRNA1825‐44, has_circRNA8493‐7, and hsa_circ_0025097. Furthermore, there were 115 down‐regulated circRNAs, such as hsa_circ_0066439, hsa_circ_0054211, hsa_circ_0095920, hsa_circ_0122984, hsa_circ_0113067, hsa_circ_0039155, hsa_circRNA4014‐45, hsa_circ_0122979, hsa_circ_0059665, and hsa_circ_0009319. The circRNAs hsa_circ_0066439, hsa_circ_0081241, and hsa_circ_0122984 can regulate multiple signal pathways to participate in the AMI process through hsa‐miR‐1254, hsa‐miR‐328‐5p, and other miRNAs. In addition, the expression of circRNA‐miRNA in peripheral blood is related to the network. Differentially expressed circRNAs are involved in chromatin organization, chromatin‐modifying enzymes, signal transduction, lysine degradation, the mitogen‐activated protein kinase (MAPK) signaling pathway, focal adhesion, and a variety of other pathways, such as myocardial infarction, coronary heart disease, hypertension, and other diseases. The gene ontology analysis results show that molecular function mainly involves binding and molecular structural activity, whereas the biological process mainly involves a single biological process, a cellular component for organization, and a cellular process, and the cellular component mainly involves a protein complex, an extracellular matrix, and a membrane.

**Conclusion:**

circRNA and microRNA interact to participate in the development of AMI. circRNA may be involved in the pathogenesis of AMI.

## Graphical abstract

650 circRNAs are differentially expressed in AMI disease, and the interaction between circRNA and miRNA is involved in the occurrence and development of AMI. Among them, hsa_circ_0066439, hsa_circ_0081241, hsa_circ_0122984, and other highly different circRNAs (hsa_circ_0043563, hsa_circ_0119137, hsa_circ_0025097, hsa_circ_0106804, hsa_circ_0085214, and hsa_circ_0028302) may regulate multiple signaling pathways by combining with miR‐328‐5p, and then participate in the pathogenesis of AMI, providing a new breakthrough point for AMI diagnosis or targeted therapy.

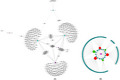

## 1 INTRODUCTION

In recent years, the incidence, disability, and mortality in cardiovascular diseases (CVDs) have increased dramatically, and the main cause of death in CVDs is acute myocardial infarction (AMI).^1^, ^2^ Although significant progress has been made in the diagnosis and treatment of AMI, effective therapeutic targets for the protection of myocardial cells from apoptosis remain limited. Therefore, there is an urgent need to understand the pathogenesis of AMI at the molecular level. Most important is the need for the discovery of novel molecular entities for AMI‐related apoptosis. Recently, noncoding RNA (ncRNA) has been suggested as a biomarker for AMI.^3^ Circular RNA (circRNA) is a type of ncRNA that exists in the form of a covalently closed continuous loop and is stably expressed in many types of cells, and its ability to regulate gene expression comes mainly from its binding or inhibiting of microRNA (miRNA).^4^, ^5^

Several studies have demonstrated the key role of circRNA in heart development and physiology.^6^ The abnormal expression of circRNA has been linked to CVDs such as heart failure, myocardial infarction, and atherosclerosis, suggesting the potential importance of circRNA in these pathological conditions.^7^, ^8^, ^9^ However, there have been few studies on the correlation between circRNA and AMI. In this study, a microarray gene chip was used to analyze the circRNA expression profile of patients with AMI, and the circRNA with a large expression difference from the control group was analyzed. By searching for candidate circRNA related to AMI, a new promising breakthrough point was provided for AMI diagnostic markers or targeted therapy, and key information was provided for revealing the complex regulatory mechanism of A‐MI.

## 2 MATERIALS AND METHODS

### 2.1 Subject selection and sample collection

Selection of study subjects: The study selected 3 patients diagnosed with AMI and 3 healthy subjects.

Inclusion and exclusion criteria：The diagnostic criteria for AMI were as follows：

1. Typical severe sternum pain with a duration of more than 30 minutes: The clinical manifestations are dull pain, squeezing pain in the posterior sternum or precordial area, and radioactive pain lasting more than 30 minutes in the neck, shoulder, and back, accompanied by sweating and dying. Furthermore, there may be clinical manifestations of heart failure or cardiogenic shock.
2. Typical dynamic changes of the patient's electrocardiogram: The patient's relevant signs were dynamically monitored by electrocardiogram, and the results of the electrocardiogram showed pathological abnormal Q waves and ST segment elevation. Although the electrocardiogram did not have pathological Q waves or ST segment elevation, it showed that the T wave and ST segment had an ischemic and dynamically changing performance.
3. Increased levels of creatinine kinase isoenzyme MB, myoglobin, and cardiac troponin I.
4. Emergency coronary angiography or percutaneous coronary intervention (PCI), confirming that at least one of the three main coronary arteries had a luminal stenosis greater than 50%.

By meeting 2 of the 4 criteria above, the diagnosis of AMI can be made.

Exclusion criteria were as follows：previous old myocardial infarction or PCI; acute or chronic infection; hematological or systemic immune diseases; severe heart, kidney, liver, and lung dysfunction; combined stroke; combined thyroid disease; previous various organ transplants; known or treated malignant tumors; no family history of other cardiovascular‐related diseases or hypertension, diabetes, etc The definition of hypertension is based on the presence of a systolic blood pressure of ≥140 mmHg and a diastolic blood pressure of ≥ 90 mmHg measured on different days or currently using antihypertensive drugs. The definition of diabetes is based on clinical characteristics and the requirements of dietary treatment or drug treatment to control blood sugar. Hyperlipidemia is defined as the total serum cholesterol level of ≥5.2 mmol/L, a triglyceride level of ≥1.7 mmol/L, or low‐density lipoprotein of ≥2.6 mmol/L, or taking statins.

The control group was a healthy population. All selected persons signed an informed consent form. All subjects had blood samples taken and combined with ethylenediaminetetraacetic acid (EDTA) anticoagulant within 3 h of admission, which were centrifuged at 3000 r/min for 10 min within 4 h, and collected plasma and centrifuged at 2000 r/min for 10 min. The separated supernatant was stored at −80°C. The study was carried out in accordance with the ethics committee of the Institute and Ethical Committees.

### 2.2 RNA extraction

The total RNA from the each patient's plasma was extracted using TRIzol reagent (Thermo Fisher Scientific, Waltham, MA, USA) according to the manufacturer's instructions. In accordance with the manufacturer's procedures, a mirVana miRNA Isolation Kit (Ambion, Austin, TX, USA) was used for purification. The purity and concentration of the RNA were determined from a 260/280 reading by using a spectrophotometer (NanoDrop Nd‐1000, Thermo Fisher Scientific, Waltham, MA, USA). RNA integrity was determined with a lab‐on‐a‐chip kit using an RNA 6000 nano chip and Bioanalyzer 2100 (Agilent Technologies, Santa Clara, CA, USA) to assess RNA quality comprehensively.

### 2.3 RNA amplification, labeling, and hybridization

Fluorescent dye Cy3 dCTP‐labeled cDNA was prepared using Eberwine linear RNA amplification and enzymatic reaction.^10^ In addition, we used a labeling kit (CapitalBio, Beijing, China) and CapitalBio‐cRNA amplification to produce higher yields of labeled cDNA according to the manufacturer's instructions.

### 2.4 Microarray imaging and data analysis

Data normalization and difference analysis were performed on the circRNA array data using GeneSpring software V13.0 (Agilent Technologies, Santa Clara, CA, USA). The data results were analyzed for data summarization, normalization, and quality control. These circRNA target sequences were obtained from Circbase, Deepbase, and Rybak‐Wolf (2015).^11^ In order to select differentially expressed genes, thresholds of > 2 and <−2‐fold changes were used in this study, and the *P*‐value corrected by the Benjamini‐Hochberg procedure was 0.05. Using the adjust data function of Cluster 3.0 (Stanford University, Stanford, CA, USA) software, the data were log2‐transformed, and the median location was centered on the gene, and then, hierarchical clustering with average linkage was used for further analysis. Finally, Java TreeView (Stanford University School of Medicine, Stanford, CA, USA) was used for tree visualization.

### 2.5 Construction of the circRNA‐miRNA network

After the differentially expressed circRNAs were screened, StarBase (Sun Yat‐sen University, China) software was used to predict the target miRNAs of the circRNAs screened in the AMI group to obtain a list of miRNAs. circRNA plays an important role in miRNA function and transcriptional control by acting as a competitive endogenous RNA or positive regulator on its parent‐encoded gene. A circRNA‐miRNA network was constructed based on miRanda database prediction (http://mirdb.org/). These circRNA‐miRNA pairs were selected to construct the network using the open source bioinformatics software Cytoscape (National Institute of General Medical Sciences, USA). In network analysis, degree centrality is defined as the link number between one node and another node. Degree is the simplest and most important measure of gene centrality in a network of relative importance.^12^

### 2.6 Enrichment analysis

The NHGRI GWAS Catalog (http://www.genome.gov/gwastudies/), KEGG DISEASE (http://www.genome.jp/kegg/disease/), and OMIM (http://www.ncbi.nlm.nih.gov/omim) bioinformatics databases were used to search for the disease enrichment analysis of the genes that were significantly expressed in the whole blood samples of the patients with AMI, and *P* < .05 was considered significant. The differentially expressed circRNAs in the whole blood of subjects were analyzed by pathway enrichment analysis using the Reactome, KEGG PATHWAY, BioCyc, and PANTHER databases, and *P* < .05 was considered as a meaningful analysis. Similarly, gene ontology analysis was performed on the linear mRNA transcripts corresponding to the 650 differentially expressed circRNAs selected from the specimens of the AMI group. The analysis included mainly molecular functions, biological processes, and cellular components.

### 2.7 Statistical analysis

Basic data were gathered regarding the age, weight, height, blood pressure, and blood lipids of the patients in the control and AMI groups. Statistical calculations were performed using SPSS (version 23.0, IBM, USA) software, and continuous data were expressed as the mean + standard deviation ($\bar{x}+s$). The t test was used to compare the continuous variables between the two groups, and the categorical variables were expressed as counts, while the chi‐square test was used to compare the categorical variables between the two groups. *P* < .05 was considered statistically significant.

## 3 RESULTS

### 3.1 Population parameters of the subjects

The population parameters of the subjects are shown in Table 1. It can be seen from this table that there were no statistically significant differences for age, body mass index, systolic blood pressure, diastolic blood pressure, or blood lipid biochemistry between the AMI group and control group (*P* > .05).

**Table 1:** Subject baseline data

Groups	n	Age (years)	BMI (kg/m2)	SBP (mmHg)	DBP (mmHg)	CHO (mmol/l)	TG (mmol/l)	HDL (mmol/l)	LDL (mmol/l)	APOA1 (g/l)	APOB (g/l)	CK‐MB (ng/ml)	MYO (ng/ml)	CTnI (ng/ml)
Control	3	47 ± 10.54	24.42 ± 1.73	86.23 ± 3.42	135.77 ± 5.57	4.14 ± 0.4	1.55 ± 0.06	0.96 ± 0.08	2.26 ± 0.45	1.05 ± 0.12	0.92 ± 0.1	1.45 ± 0.27	50.68 ± 26.28	0.04 ± 0.02
AMI	3	45.67 ± 9.29	25.74 ± 1.21	84.90 ± 4.56	132.9 ± 3.96	3.98 ± 0.23	1.66 ± 0.06	0.93 ± 0.05	2.41 ± 0.38	1.0 ± 0.11	0.75 ± 0.08	14.51 ± 7.15*	424.78 ± 207.35*	12.86 ± 6.68*

AMI, acute myocardial infarction; APOA1, apolipoprotein A1; APOB, apolipoprotein B; BMI, body mass index; CHO, cholesterol; CK‐MB, creatine kinase isoenzyme MB; CTnI, cardiac troponin I; DBP, diastolic blood pressure; HDL, high‐density lipoprotein; LDL, low‐density lipoprotein; MYO, myoglobin; SBP, systolic blood pressure; TG, triglyceride.

* *P* < .05.

### 3.2 Microarray gene chip analysis identified significantly different circRNAs

**Figure 1:**
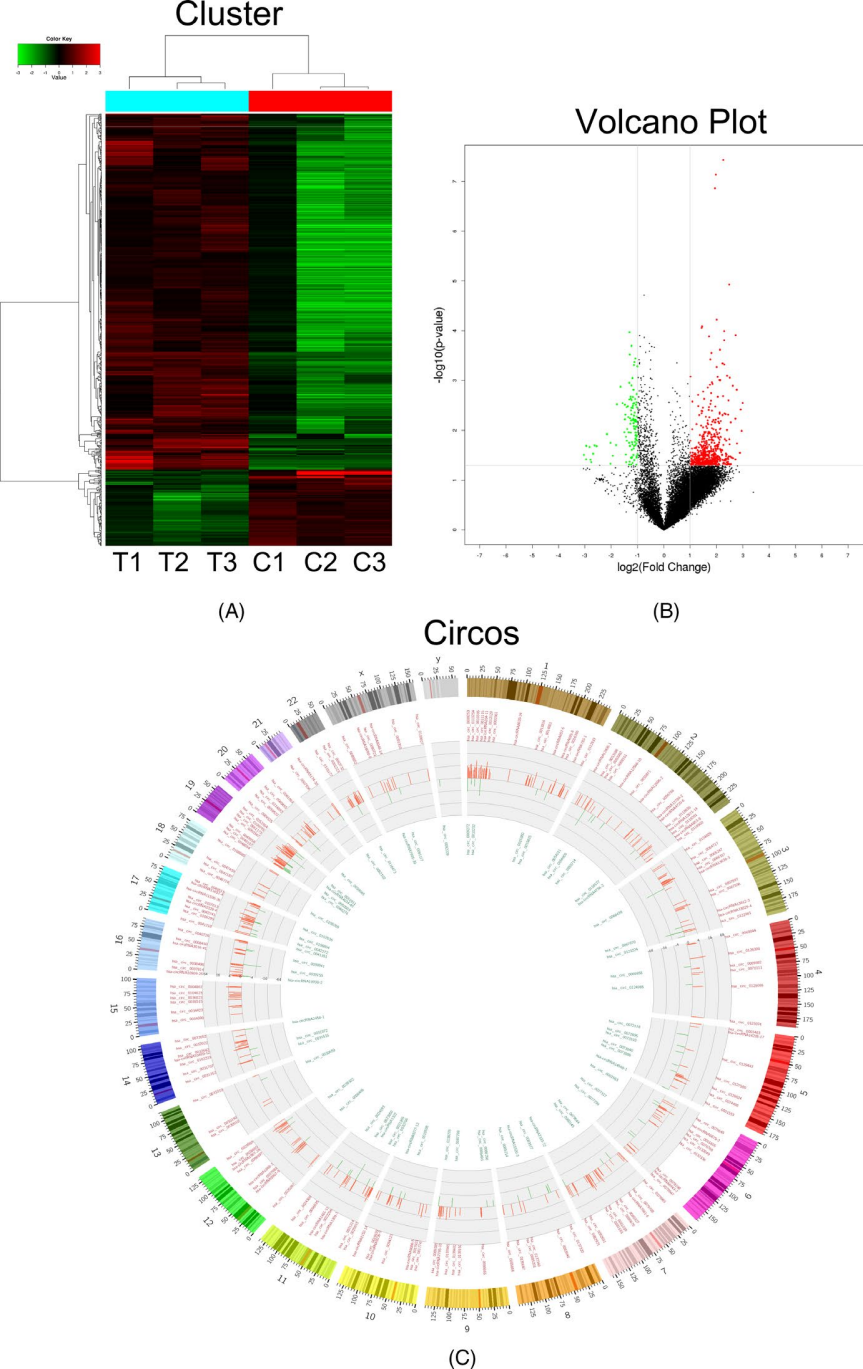
Profiling of circular RNAs in the plasmas from AMI patients and normal controls. (a) Cluster analysis of gene expression between AMI patients and control samples. Each column represents the expression profile of a tissue sample, and each row corresponds to a circRNA. High expression level is indicated by red and lower levels by green. (b) Volcano plot shows the up‐regulated and down‐regulated circRNAs in AMI patients vs control group. Higher expression levels are indicated by red, lower expression levels are indicated by green, and no significant difference is indicated by black. (c) Circos diagram of the difference circRNA between the control group and the AMI group. The length of the bar indicates the multiple of the differential gene, the red bar in the inner circle indicates that the differential gene is up‐regulated, and the green bar indicates that the differential gene is down‐regulated

In order to understand the molecular mechanisms involved and to search for AMI biomarkers, a microarray gene chip was used to screen and analyze the circRNA expression profiles of our study. A total of 13 804 circRNAs were identified through the gene chip analysis of circRNA expression profiles (Figure 1A, Figure 1B). As compared to the control group, 650 circRNAs were screened for differential expression in the AMI group (Figure 1B), among which 535 circRNAs were up‐regulated (the top ten up‐regulated circRNAs were as follows: hsa_circ_0050908, hsa_circRNA4010‐22, hsa_circ_0081241, hsa_circ_0010551, hsa_circRNA4010‐20, hsa_circRNA14702, hsa_circ_0115392, has_circRNA1825‐44, has_circRNA8493‐7, and hsa_circ_0025097) and 115 circRNAs were down‐regulated (the top ten down‐regulated circRNAs were as follows: hsa_circ_0066439, hsa_circ_0054211, hsa_circ_0095920, hsa_circ_0122984, hsa_circ_0113067, hsa_circ_0039155, hsa_circRNA4014‐45, hsa_circ_0122979, hsa_circ_0059665, and hsa_circ_0009319). It can be seen that circRNA may play a certain role in the occurrence and progression of AMI (Table 2, Figure 1C).

**Table 2:** Differentially expressed circRNAs between AMI group and control group

Number	ProbeName	*P*	FC (abs)	Regulation	Chromosome	miRNA number more than 2
1	hsa_circ_0050908	.0028	7.9732	up	chr19	‐
2	hsa_circRNA4010‐22	.0102	7.7393	up	chr19	2
3	hsa_circ_0081241	.0038	7.5621	up	chr7	8
4	hsa_circ_0010551	.0283	7.4077	up	chr1	100
5	hsa_circRNA4010‐20	.0182	6.8538	up	chr19	‐
6	hsa_circRNA14702	.0015	6.8083	up	chr6	‐
7	hsa_circ_0115392	.0001	6.6244	up	chr20	‐
8	hsa_circRNA1825‐44	.0383	6.5754	up	chr12	1
9	hsa_circRNA8493‐7	.0058	6.5741	up	chr1	‐
10	hsa_circ_0025097	.0361	6.1700	up	chr12	100
11	hsa_circ_0097682	.0013	6.1357	up	chr12	‐
12	hsa_circ_0003531	.0352	5.9584	up	chr3	‐
13	hsa_circ_0049121	.0474	5.8384	up	chr19	3
14	hsa_circ_0060784	.0069	5.8181	up	chr20	17
15	hsa_circ_0044537	.0334	5.7443	up	chr17	86
16	hsa_circ_0016320	.0488	5.7141	up	chr1	100
17	hsa_circ_0015063	.0009	5.6756	up	chr1	10
18	hsa_circ_0050906	.0191	5.6475	up	chr19	‐
19	hsa_circ_0135204	.0151	5.6078	up	chr7	8
20	hsa_circ_0020887	.0000	5.5918	up	chr11	26
21	hsa_circ_0126393	.0468	5.5484	up	chr4	‐
22	hsa_circ_0000946	.0333	5.4939	up	chr19	100
23	hsa_circ_0080478	.0025	5.4834	up	chr7	75
24	hsa_circ_0075752	.0378	5.4398	up	chr6	10
25	hsa_circ_0118836	.0048	5.4305	up	chr2	‐
26	hsa_circ_0074348	.0478	5.4190	up	chr5	‐
27	hsa_circ_0114870	.0424	5.4090	up	chr20	1
28	hsa_circ_0043563	.0357	5.4019	up	chr17	54
29	hsa_circ_0081150	.0458	5.3873	up	chr7	16
30	hsa_circ_0114865	.0432	5.3303	up	chr20	2
31	hsa_circ_0096364	.0486	5.3301	up	chr11	16
32	hsa_circRNA12871‐14	.0008	5.2871	up	chr2	‐
33	hsa_circ_0007326	.0098	5.2759	up	chr14	2
34	hsa_circ_0115190	.0438	5.2530	up	chr20	17
35	hsa_circ_0041218	.0487	5.2061	up	chr17	90
36	hsa_circRNA5200‐8	.0372	5.1911	up	chr21	4
37	hsa_circRNA5199‐28	.0303	5.1872	up	chr21	2
38	hsa_circ_0026362	.0161	5.1638	up	chr12	‐
39	hsa_circ_0000542	.0329	5.1468	up	chr14	2
40	hsa_circ_0004867	.0293	5.1261	up	chr15	91
41	hsa_circ_0041635	.0398	5.1003	up	chr17	40
42	hsa_circ_0026432	.0047	5.0848	up	chr12	1
43	hsa_circ_0081616	.0375	5.0751	up	chr7	100
44	hsa_circRNA12789‐2	.0468	4.9916	up	chr2	‐
45	hsa_circ_0016846	.0150	4.9841	up	chr1	6
46	hsa_circ_0058306	.0385	4.9675	up	chr2	33
47	hsa_circRNA7287‐4	.0460	4.9535	up	chr7	1
48	hsa_circ_0017921	.0045	4.9350	up	chr10	9
49	hsa_circRNA12245‐5	.0005	4.9070	up	chr19	9
50	hsa_circ_0110254	.0002	4.8930	up	chr1	2
51	hsa_circRNA12245‐25	.0001	4.8891	up	chr19	6
52	hsa_circ_0061943	.0402	4.8853	up	chr21	35
53	hsa_circ_0060832	.0449	4.8002	up	chr20	11
54	hsa_circRNA5599‐5	.0197	4.7854	up	chr3	‐
55	hsa_circ_0028650	.0000	4.7764	up	chr12	11
56	hsa_circ_0029375	.0467	4.7582	up	chr12	5
57	hsa_circRNA9376‐69	.0021	4.7539	up	chr11	9
58	hsa_circRNA5060‐14	.0049	4.6888	up	chr20	‐
59	hsa_circ_0009590	.0005	4.6848	up	chr1	‐
60	hsa_circ_0117770	.0391	4.6756	up	chr2	100
61	hsa_circ_0054098	.0250	4.6417	up	chr2	11
62	hsa_circ_0007897	.0312	4.6374	up	chr3	6
63	hsa_circ_0119137	.0308	4.6289	up	chr2	100
64	hsa_circ_0081635	.0247	4.5833	up	chr7	‐
65	hsa_circ_0122013	.0274	4.5272	up	chr3	4
66	hsa_circ_0044563	.0137	4.4890	up	chr17	69
67	hsa_circ_0115393	.0065	4.4798	up	chr20	6
68	hsa_circ_0053188	.0253	4.4770	up	chr2	100
69	hsa_circ_0050905	.0134	4.4564	up	chr19	‐
70	hsa_circ_0063356	.0092	4.4373	up	chr22	16
71	hsa_circ_0079065	.0373	4.4277	up	chr7	3
72	hsa_circRNA8426‐8	.0404	4.4039	up	chr1	12
73	hsa_circ_0124205	.0010	4.4011	up	chr3	‐
74	hsa_circ_0044521	.0237	4.3930	up	chr17	100
75	hsa_circ_0074574	.0010	4.3928	up	chr5	90
76	hsa_circ_0026092	.0002	4.3773	up	chr12	100
77	hsa_circRNA11596‐26	.0095	4.3764	up	chr17	2
78	hsa_circ_0109687	.0410	4.3675	up	chr19	100
79	hsa_circ_0050237	.0357	4.3469	up	chr19	21
80	hsa_circRNA2312‐10	.0044	4.3465	up	chr14	2
81	hsa_circ_0102224	.0046	4.3441	up	chr14	5
82	hsa_circ_0067036	.0154	4.3368	up	chr3	‐
83	hsa_circRNA3597‐4	.0113	4.3000	up	chr18	1
84	hsa_circ_0050900	.0010	4.2987	up	chr19	4
85	hsa_circ_0080676	.0342	4.2818	up	chr7	‐
86	hsa_circ_0007935	.0175	4.2791	up	chr2	‐
87	hsa_circ_0088235	.0071	4.2782	up	chr9	12
88	hsa_circ_0004847	.0482	4.2735	up	chr12	9
89	hsa_circ_0045961	.0475	4.2424	up	chr17	36
90	hsa_circRNA12308‐1	.0142	4.2413	up	chr19	1
91	hsa_circRNA4720‐6	.0468	4.2333	up	chr2	1
92	hsa_circ_0021922	.0384	4.2236	up	chr11	8
93	hsa_circ_0056766	.0490	4.2095	up	chr2	‐
94	hsa_circRNA3313‐7	.0193	4.2026	up	chr17	11
95	hsa_circ_0068491	.0193	4.2000	up	chr3	8
96	hsa_circ_0035515	.0067	4.1976	up	chr15	27
97	hsa_circ_0063873	.0057	4.1935	up	chr22	‐
98	hsa_circ_0115275	.0136	4.1572	up	chr20	100
99	hsa_circ_0031357	.0210	4.1505	up	chr14	5
100	hsa_circ_0116655	.0377	4.1444	up	chr22	36
101	hsa_circ_0132805	.0498	4.1399	up	chr7	‐
102	hsa_circRNA8620‐24	.0324	4.1352	up	chr1	‐
103	hsa_circ_0114892	.0021	4.1320	up	chr20	100
104	hsa_circ_0031252	.0235	4.1315	up	chr14	‐
105	hsa_circ_0040794	.0008	4.1162	up	chr16	100
106	hsa_circ_0081548	.0341	4.1045	up	chr7	9
107	hsa_circ_0102223	.0194	4.1004	up	chr14	‐
108	hsa_circ_0091005	.0228	4.0970	up	chrX	51
109	hsa_circ_0078460	.0415	4.0936	up	chr6	3
110	hsa_circRNA11596‐28	.0119	4.0882	up	chr17	18
111	hsa_circ_0106756	.0202	4.0860	up	chr17	‐
112	hsa_circ_0034918	.0431	4.0830	up	chr15	6
113	hsa_circ_0035020	.0347	4.0708	up	chr15	7
114	hsa_circ_0106684	.0364	4.0636	up	chr17	5
115	hsa_circ_0010572	.0342	4.0474	up	chr1	100
116	hsa_circ_0093278	.0407	4.0439	up	chr10	‐
117	hsa_circ_0093277	.0396	4.0366	up	chr10	2
118	hsa_circ_0027083	.0424	4.0295	up	chr12	1
119	hsa_circ_0088229	.0001	4.0238	up	chr9	38
120	hsa_circRNA1309‐3	.0005	4.0215	up	chr11	‐
121	hsa_circRNA2312‐5	.0109	4.0103	up	chr14	‐
122	hsa_circ_0039003	.0495	3.9968	up	chr16	14
123	hsa_circ_0043159	.0358	3.9846	up	chr17	22
124	hsa_circ_0065957	.0203	3.9833	up	chr3	4
125	hsa_circ_0046774	.0461	3.9730	up	chr18	100
126	hsa_circ_0037814	.0360	3.9721	up	chr16	‐
127	hsa_circ_0032221	.0346	3.9681	up	chr14	100
128	hsa_circRNA797‐8	.0257	3.9445	up	chr1	7
129	hsa_circ_0051640	.0412	3.9368	up	chr19	10
130	hsa_circ_0062530	.0491	3.9361	up	chr22	25
131	hsa_circ_0093276	.0311	3.9357	up	chr10	3
132	hsa_circ_0104623	.0098	3.9353	up	chr15	17
133	hsa_circ_0133736	.0000	3.9342	up	chr7	‐
134	hsa_circ_0137340	.0239	3.9308	up	chr8	100
135	hsa_circRNA16090‐8	.0462	3.9278	up	chrX	‐
136	hsa_circ_0024527	.0452	3.9168	up	chr11	16
137	hsa_circRNA11985‐8	.0061	3.9155	up	chr19	4
138	hsa_circ_0050588	.0455	3.9151	up	chr19	‐
139	hsa_circ_0089115	.0349	3.9131	up	chr9	14
140	hsa_circ_0060538	.0355	3.9022	up	chr20	2
141	hsa_circ_0045044	.0135	3.8987	up	chr17	10
142	hsa_circ_0044532	.0437	3.8951	up	chr17	100
143	hsa_circ_0052364	.0069	3.8833	up	chr19	‐
144	hsa_circ_0049107	.0343	3.8821	up	chr19	100
145	hsa_circRNA3264‐5	.0033	3.8722	up	chr17	‐
146	hsa_circ_0082921	.0297	3.8617	up	chr7	30
147	hsa_circ_0135667	.0228	3.8598	up	chr8	1
148	hsa_circ_0091310	.0389	3.8561	up	chrX	100
149	hsa_circ_0029569	.0460	3.8559	up	chr12	53
150	hsa_circ_0002849	.0134	3.8403	up	chr14	28
151	hsa_circ_0028516	.0473	3.8380	up	chr12	100
152	hsa_circRNA11054‐14	.0208	3.8377	up	chr16	1
153	hsa_circRNA13583‐5	.0000	3.8372	up	chr3	‐
154	hsa_circ_0059527	.0451	3.8360	up	chr20	6
155	hsa_circ_0054140	.0287	3.8258	up	chr2	‐
156	hsa_circ_0109852	.0128	3.8251	up	chr19	100
157	hsa_circ_0125974	.0069	3.8101	up	chr4	‐
158	hsa_circ_0036023	.0375	3.8092	up	chr15	8
159	hsa_circ_0090862	.0414	3.8054	up	chrX	2
160	hsa_circRNA9907‐9	.0364	3.7986	up	chr12	100
161	hsa_circ_0008101	.0304	3.7936	up	chr2	5
162	hsa_circ_0009253	.0359	3.7920	up	chr1	‐
163	hsa_circRNA5242‐25	.0423	3.7915	up	chr22	56
164	hsa_circRNA1670‐5	.0386	3.7898	up	chr12	‐
165	hsa_circ_0134796	.0119	3.7869	up	chr7	1
166	hsa_circ_0051230	.0179	3.7867	up	chr19	1
167	hsa_circ_0065947	.0473	3.7845	up	chr3	47
168	hsa_circ_0132049	.0290	3.7667	up	chr6	‐
169	hsa_circ_0068342	.0314	3.7577	up	chr3	100
170	hsa_circ_0005237	.0376	3.7485	up	chr12	3
171	hsa_circ_0089852	.0388	3.7423	up	chrX	‐
172	hsa_circRNA3967‐8	.0132	3.7356	up	chr19	4
173	hsa_circ_0033552	.0394	3.7291	up	chr14	80
174	hsa_circ_0032609	.0382	3.7151	up	chr14	28
175	hsa_circ_0078198	.0339	3.7122	up	chr6	41
176	hsa_circ_0022296	.0262	3.7103	up	chr11	23
177	hsa_circRNA3864‐4	.0471	3.6980	up	chr19	25
178	hsa_circRNA2644‐16	.0062	3.6964	up	chr15	28
179	hsa_circ_0132343	.0095	3.6932	up	chr6	100
180	hsa_circ_0041099	.0158	3.6809	up	chr16	100
181	hsa_circ_0047459	.0241	3.6796	up	chr18	100
182	hsa_circ_0105164	.0324	3.6692	up	chr16	18
183	hsa_circ_0079068	.0303	3.6599	up	chr7	3
184	hsa_circ_0126390	.0264	3.6444	up	chr4	‐
185	hsa_circ_0014206	.0433	3.6420	up	chr1	28
186	hsa_circRNA12408‐1	.0383	3.6188	up	chr2	4
187	hsa_circ_0011244	.0159	3.6126	up	chr1	‐
188	hsa_circRNA13829‐4	.0012	3.6085	up	chr3	‐
189	hsa_circ_0038498	.0367	3.6079	up	chr16	‐
190	hsa_circ_0057189	.0454	3.6013	up	chr2	‐
191	hsa_circ_0109756	.0306	3.5994	up	chr19	100
192	hsa_circ_0035705	.0441	3.5992	up	chr15	100
193	hsa_circRNA12566‐10	.0408	3.5987	up	chr2	5
194	hsa_circ_0139927	.0067	3.5914	up	chrX	11
195	hsa_circ_0081593	.0136	3.5912	up	chr7	1
196	hsa_circRNA12696‐2	.0405	3.5862	up	chr2	24
197	hsa_circ_0051173	.0392	3.5726	up	chr19	56
198	hsa_circ_0032642	.0487	3.5663	up	chr14	1
199	hsa_circRNA2921‐20	.0151	3.5602	up	chr16	1
200	hsa_circ_0010191	.0090	3.5588	up	chr1	20
201	hsa_circ_0113430	.0011	3.5507	up	chr1	10
202	hsa_circ_0034090	.0040	3.5492	up	chr15	‐
203	hsa_circRNA7202‐21	.0484	3.5251	up	chr7	100
204	hsa_circ_0063931	.0476	3.5249	up	chr22	100
205	hsa_circ_0008450	.0443	3.5224	up	chr16	5
206	hsa_circ_0058447	.0410	3.5211	up	chr2	1
207	hsa_circRNA13061‐2	.0176	3.5168	up	chr20	25
208	hsa_circ_0075645	.0353	3.5130	up	chr6	3
209	hsa_circ_0002720	.0464	3.5129	up	chr1	6
210	hsa_circ_0113486	.0140	3.5110	up	chr1	4
211	hsa_circ_0124829	.0479	3.5061	up	chr3	13
212	hsa_circ_0036904	.0449	3.5045	up	chr15	3
213	hsa_circ_0016796	.0488	3.5024	up	chr1	7
214	hsa_circ_0051639	.0003	3.4975	up	chr19	10
215	hsa_circ_0057402	.0314	3.4962	up	chr2	15
216	hsa_circRNA9086‐5	.0122	3.4923	up	chr10	‐
217	hsa_circ_0104381	.0266	3.4913	up	chr15	‐
218	hsa_circRNA11981‐18	.0077	3.4870	up	chr19	52
219	hsa_circ_0081161	.0433	3.4850	up	chr7	37
220	hsa_circ_0052788	.0251	3.4820	up	chr2	2
221	hsa_circ_0112353	.0232	3.4723	up	chr1	‐
222	hsa_circ_0037423	.0018	3.4716	up	chr16	75
223	hsa_circRNA8046‐14	.0137	3.4681	up	chrX	51
224	hsa_circ_0031707	.0154	3.4672	up	chr14	19
225	hsa_circ_0060455	.0025	3.4601	up	chr20	2
226	hsa_circRNA3930‐2	.0414	3.4389	up	chr19	7
227	hsa_circRNA6462	.0394	3.4387	up	chr5	1
228	hsa_circ_0026618	.0213	3.4371	up	chr12	29
229	hsa_circ_0050367	.0472	3.4357	up	chr19	8
230	hsa_circ_0049457	.0438	3.4170	up	chr19	18
231	hsa_circ_0080798	.0029	3.4043	up	chr7	2
232	hsa_circ_0048975	.0454	3.3981	up	chr19	54
233	hsa_circRNA7061‐6	.0004	3.3933	up	chr7	‐
234	hsa_circ_0050183	.0482	3.3794	up	chr19	15
235	hsa_circRNA8934‐1	.0472	3.3781	up	chr1	2
236	hsa_circ_0099199	.0477	3.3731	up	chr12	100
237	hsa_circ_0070111	.0447	3.3692	up	chr4	7
238	hsa_circ_0020753	.0483	3.3629	up	chr11	43
239	hsa_circ_0094123	.0395	3.3626	up	chr10	‐
240	hsa_circ_0007436	.0012	3.3565	up	chr16	8
241	hsa_circ_0013819	.0114	3.3526	up	chr1	100
242	hsa_circ_0039006	.0434	3.3509	up	chr16	‐
243	hsa_circRNA11853‐7	.0497	3.3415	up	chr19	2
244	hsa_circRNA1868‐3	.0265	3.3342	up	chr12	‐
245	hsa_circ_0046208	.0261	3.3328	up	chr17	‐
246	hsa_circ_0079357	.0108	3.3212	up	chr7	7
247	hsa_circ_0049030	.0405	3.3192	up	chr19	28
248	hsa_circ_0063241	.0356	3.3151	up	chr22	16
249	hsa_circ_0001463	.0284	3.3150	up	chr5	3
250	hsa_circ_0050824	.0054	3.3146	up	chr19	17
251	hsa_circRNA12060‐3	.0489	3.2955	up	chr19	6
252	hsa_circ_0024576	.0195	3.2948	up	chr11	7
253	hsa_circ_0000775	.0467	3.2901	up	chr17	4
254	hsa_circ_0124075	.0001	3.2883	up	chr3	1
255	hsa_circ_0078647	.0403	3.2858	up	chr6	5
256	hsa_circ_0010395	.0403	3.2858	up	chr1	68
257	hsa_circRNA6943‐7	.0154	3.2839	up	chr6	1
258	hsa_circ_0024308	.0034	3.2758	up	chr11	100
259	hsa_circRNA4853‐8	.0266	3.2729	up	chr2	100
260	hsa_circ_0021075	.0260	3.2701	up	chr11	21
261	hsa_circ_0064717	.0333	3.2648	up	chr3	3
262	hsa_circ_0102265	.0437	3.2622	up	chr14	39
263	hsa_circ_0116219	.0039	3.2562	up	chr22	4
264	hsa_circ_0009410	.0308	3.2540	up	chr1	70
265	hsa_circ_0011115	.0106	3.2510	up	chr1	‐
266	hsa_circ_0090418	.0418	3.2438	up	chrX	48
267	hsa_circ_0044107	.0328	3.2404	up	chr17	‐
268	hsa_circRNA15745‐14	.0417	3.2342	up	chr9	100
269	hsa_circ_0066317	.0440	3.2315	up	chr3	46
270	hsa_circRNA482‐153	.0470	3.2287	up	chr1	2
271	hsa_circRNA7202‐26	.0115	3.2260	up	chr7	100
272	hsa_circ_0032569	.0468	3.2199	up	chr14	7
273	hsa_circ_0023203	.0116	3.2154	up	chr11	100
274	hsa_circ_0113354	.0480	3.2153	up	chr1	11
275	hsa_circ_0044525	.0075	3.2145	up	chr17	74
276	hsa_circ_0049587	.0402	3.2077	up	chr19	3
277	hsa_circ_0009768	.0457	3.1982	up	chr1	4
278	hsa_circ_0137665	.0454	3.1963	up	chr9	18
279	hsa_circ_0021214	.0265	3.1851	up	chr11	4
280	hsa_circRNA1075‐40	.0014	3.1817	up	chr10	11
281	hsa_circ_0085201	.0063	3.1787	up	chr8	100
282	hsa_circ_0032592	.0315	3.1777	up	chr14	23
283	hsa_circ_0080327	.0213	3.1748	up	chr7	30
284	hsa_circ_0049973	.0071	3.1691	up	chr19	100
285	hsa_circRNA1828‐19	.0370	3.1664	up	chr12	‐
286	hsa_circ_0138057	.0428	3.1594	up	chr9	1
287	hsa_circ_0009823	.0323	3.1594	up	chr1	92
288	hsa_circRNA3136‐8	.0266	3.1546	up	chr16	20
289	hsa_circ_0039874	.0129	3.1510	up	chr16	100
290	hsa_circRNA9236‐9	.0455	3.1500	up	chr10	3
291	hsa_circRNA1456‐53	.0383	3.1446	up	chr11	35
292	hsa_circ_0089150	.0383	3.1376	up	chr9	‐
293	hsa_circ_0119201	.0090	3.1290	up	chr2	3
294	hsa_circ_0048322	.0315	3.1262	up	chr19	74
295	hsa_circ_0089419	.0303	3.1240	up	chr9	50
296	hsa_circRNA15570‐43	.0232	3.1226	up	chr8	1
297	hsa_circ_0082601	.0468	3.1221	up	chr7	14
298	hsa_circ_0012345	.0378	3.1087	up	chr1	13
299	hsa_circRNA204‐11	.0179	3.1038	up	chr1	3
300	hsa_circ_0109858	.0194	3.0877	up	chr19	100
301	hsa_circRNA1967‐24	.0163	3.0838	up	chr12	12
302	hsa_circ_0002361	.0443	3.0746	up	chr1	5
303	hsa_circ_0064011	.0384	3.0705	up	chr22	55
304	hsa_circ_0125089	.0454	3.0684	up	chr4	18
305	hsa_circ_0014179	.0403	3.0683	up	chr1	28
306	hsa_circRNA11842‐6	.0441	3.0676	up	chr18	100
307	hsa_circ_0029011	.0478	3.0640	up	chr12	100
308	hsa_circ_0019939	.0083	3.0502	up	chr10	100
309	hsa_circRNA4074‐19	.0219	3.0489	up	chr19	33
310	hsa_circ_0032924	.0479	3.0375	up	chr14	2
311	hsa_circ_0076661	.0371	3.0314	up	chr6	71
312	hsa_circ_0032535	.0166	3.0295	up	chr14	‐
313	hsa_circ_0106290	.0053	3.0185	up	chr17	27
314	hsa_circ_0032922	.0426	3.0154	up	chr14	3
315	hsa_circ_0055326	.0273	3.0146	up	chr2	‐
316	hsa_circ_0051724	.0270	3.0116	up	chr19	100
317	hsa_circ_0046067	.0224	3.0100	up	chr17	‐
318	hsa_circ_0078421	.0107	3.0087	up	chr6	78
319	hsa_circ_0113323	.0309	3.0039	up	chr1	100
320	hsa_circ_0088488	.0334	2.9938	up	chr9	11
321	hsa_circ_0089385	.0320	2.9935	up	chr9	9
322	hsa_circ_0112819	.0411	2.9922	up	chr1	33
323	hsa_circ_0079201	.0480	2.9902	up	chr7	12
324	hsa_circ_0005692	.0483	2.9871	up	chr16	28
325	hsa_circ_0049615	.0417	2.9843	up	chr19	100
326	hsa_circ_0044558	.0469	2.9821	up	chr17	100
327	hsa_circ_0083141	.0203	2.9802	up	chr7	97
328	hsa_circ_0085926	.0415	2.9795	up	chr8	‐
329	hsa_circ_0012128	.0453	2.9794	up	chr1	100
330	hsa_circ_0063584	.0294	2.9694	up	chr22	‐
331	hsa_circ_0047187	.0471	2.9659	up	chr18	‐
332	hsa_circ_0063534	.0439	2.9570	up	chr22	2
333	hsa_circ_0107013	.0262	2.9566	up	chr17	100
334	hsa_circ_0085866	.0180	2.9551	up	chr8	100
335	hsa_circ_0106833	.0230	2.9547	up	chr17	33
336	hsa_circRNA3926	.0465	2.9492	up	chr19	‐
337	hsa_circ_0037268	.0440	2.9451	up	chr16	73
338	hsa_circ_0063488	.0113	2.9335	up	chr22	100
339	hsa_circ_0092100	.0489	2.9307	up	chrX	‐
340	hsa_circ_0007556	.0252	2.9251	up	chr21	‐
341	hsa_circ_0056913	.0293	2.9249	up	chr2	2
342	hsa_circ_0065403	.0240	2.9227	up	chr3	100
343	hsa_circ_0044535	.0225	2.9157	up	chr17	100
344	hsa_circRNA4024‐3	.0107	2.9113	up	chr19	1
345	hsa_circ_0085882	.0444	2.9109	up	chr8	47
346	hsa_circRNA2312‐13	.0500	2.9028	up	chr14	‐
347	hsa_circ_0048054	.0358	2.9028	up	chr19	7
348	hsa_circ_0051507	.0274	2.8966	up	chr19	3
349	hsa_circ_0109795	.0417	2.8938	up	chr19	13
350	hsa_circ_0089417	.0147	2.8914	up	chr9	1
351	hsa_circRNA1246	.0482	2.8892	up	chr11	‐
352	hsa_circ_0106385	.0023	2.8866	up	chr17	29
353	hsa_circ_0050895	.0226	2.8860	up	chr19	‐
354	hsa_circ_0126795	.0424	2.8852	up	chr4	7
355	hsa_circRNA11889‐2	.0326	2.8845	up	chr19	10
356	hsa_circ_0017009	.0167	2.8702	up	chr1	14
357	hsa_circRNA1152‐14	.0305	2.8674	up	chr10	7
358	hsa_circRNA3717‐2	.0199	2.8625	up	chr18	‐
359	hsa_circ_0037270	.0323	2.8606	up	chr16	‐
360	hsa_circRNA3016‐41	.0476	2.8596	up	chr16	3
361	hsa_circ_0014801	.0292	2.8555	up	chr1	63
362	hsa_circ_0045705	.0385	2.8514	up	chr17	6
363	hsa_circ_0063527	.0132	2.8504	up	chr22	9
364	hsa_circ_0049724	.0292	2.8487	up	chr19	16
365	hsa_circ_0034423	.0458	2.8454	up	chr15	100
366	hsa_circRNA9471‐8	.0335	2.8437	up	chr11	87
367	hsa_circRNA15152‐25	.0493	2.8436	up	chr7	6
368	hsa_circRNA10499‐12	.0121	2.8419	up	chr14	17
369	hsa_circ_0055871	.0272	2.8411	up	chr2	‐
370	hsa_circRNA3521‐29	.0429	2.8394	up	chr17	26
371	hsa_circ_0074315	.0494	2.8392	up	chr5	100
372	hsa_circRNA2786‐7	.0456	2.8366	up	chr16	8
373	hsa_circ_0001223	.0264	2.8360	up	chr22	2
374	hsa_circ_0052235	.0460	2.8341	up	chr19	100
375	hsa_circ_0048074	.0205	2.8282	up	chr19	100
376	hsa_circ_0095664	.0381	2.8270	up	chr11	4
377	hsa_circ_0096835	.0017	2.8165	up	chr11	‐
378	hsa_circ_0108880	.0289	2.8109	up	chr18	100
379	hsa_circ_0060072	.0087	2.8067	up	chr20	19
380	hsa_circRNA15751‐7	.0380	2.8061	up	chr9	5
381	hsa_circRNA657‐6	.0351	2.8049	up	chr1	7
382	hsa_circ_0016316	.0247	2.8004	up	chr1	13
383	hsa_circ_0030010	.0273	2.7993	up	chr13	9
384	hsa_circ_0078702	.0035	2.7986	up	chr6	41
385	hsa_circ_0010754	.0387	2.7984	up	chr1	1
386	hsa_circ_0020615	.0369	2.7888	up	chr11	3
387	hsa_circ_0032237	.0496	2.7866	up	chr14	2
388	hsa_circRNA15950‐4	.0136	2.7659	up	chr9	14
389	hsa_circ_0067300	.0320	2.7637	up	chr3	14
390	hsa_circRNA13222‐1	.0452	2.7622	up	chr21	‐
391	hsa_circ_0016063	.0374	2.7604	up	chr1	7
392	hsa_circRNA11054‐15	.0149	2.7584	up	chr16	3
393	hsa_circRNA4223‐6	.0041	2.7582	up	chr19	1
394	hsa_circ_0087060	.0417	2.7564	up	chr9	18
395	hsa_circ_0059325	.0217	2.7513	up	chr20	1
396	hsa_circRNA1377	.0130	2.7473	up	chr11	40
397	hsa_circ_0083940	.0441	2.7468	up	chr8	100
398	hsa_circRNA5357‐12	.0387	2.7467	up	chr22	100
399	hsa_circ_0090721	.0458	2.7458	up	chrX	62
400	hsa_circ_0010714	.0490	2.7342	up	chr1	60
401	hsa_circ_0018975	.0001	2.7339	up	chr10	31
402	hsa_circ_0017743	.0226	2.7294	up	chr10	‐
403	hsa_circ_0135200	.0447	2.7221	up	chr7	7
404	hsa_circ_0057366	.0299	2.7191	up	chr2	100
405	hsa_circ_0086865	.0169	2.7160	up	chr9	100
406	hsa_circ_0027204	.0211	2.7136	up	chr12	100
407	hsa_circ_0042741	.0488	2.7118	up	chr17	10
408	hsa_circRNA11231‐9	.0314	2.7094	up	chr16	2
409	hsa_circ_0139402	.0302	2.7013	up	chr9	17
410	hsa_circRNA14685	.0482	2.6995	up	chr6	6
411	hsa_circRNA10909‐25	.0045	2.6989	up	chr16	9
412	hsa_circ_0001281	.0001	2.6967	up	chr3	2
413	hsa_circ_0076072	.0041	2.6910	up	chr6	10
414	hsa_circ_0124246	.0454	2.6868	up	chr3	3
415	hsa_circ_0065729	.0444	2.6759	up	chr3	‐
416	hsa_circRNA13812‐3	.0361	2.6685	up	chr3	‐
417	hsa_circ_0074496	.0282	2.6658	up	chr5	100
418	hsa_circ_0064827	.0080	2.6642	up	chr3	15
419	hsa_circ_0129443	.0199	2.6564	up	chr5	100
420	hsa_circ_0044163	.0451	2.6486	up	chr17	100
421	hsa_circ_0040983	.0316	2.6461	up	chr16	1
422	hsa_circ_0097672	.0258	2.6430	up	chr12	‐
423	hsa_circRNA5250‐15	.0444	2.6296	up	chr22	66
424	hsa_circ_0134538	.0384	2.6269	up	chr7	100
425	hsa_circ_0067163	.0491	2.6204	up	chr3	9
426	hsa_circ_0076768	.0448	2.6173	up	chr6	‐
427	hsa_circ_0037342	.0484	2.6162	up	chr16	50
428	hsa_circ_0053992	.0398	2.6155	up	chr2	26
429	hsa_circ_0109585	.0193	2.5950	up	chr19	1
430	hsa_circ_0016973	.0386	2.5878	up	chr1	5
431	hsa_circ_0046114	.0464	2.5858	up	chr17	84
432	hsa_circ_0065709	.0124	2.5807	up	chr3	39
433	hsa_circ_0059966	.0431	2.5687	up	chr20	23
434	hsa_circRNA14235‐17	.0454	2.5685	up	chr5	21
435	hsa_circ_0137320	.0483	2.5601	up	chr8	2
436	hsa_circRNA12330‐1	.0326	2.5564	up	chr19	3
437	hsa_circ_0046066	.0395	2.5556	up	chr17	‐
438	hsa_circRNA10194‐72	.0314	2.5552	up	chr12	81
439	hsa_circ_0122981	.0398	2.5538	up	chr3	6
440	hsa_circRNA2731‐1	.0374	2.5514	up	chr15	1
441	hsa_circ_0082648	.0413	2.5424	up	chr7	‐
442	hsa_circ_0116177	.0352	2.5420	up	chr22	2
443	hsa_circ_0116532	.0049	2.5396	up	chr22	23
444	hsa_circ_0051082	.0450	2.5327	up	chr19	1
445	hsa_circ_0063612	.0455	2.5325	up	chr22	100
446	hsa_circ_0069044	.0475	2.5302	up	chr4	2
447	hsa_circ_0081609	.0362	2.5222	up	chr7	100
448	hsa_circ_0127589	.0335	2.5174	up	chr5	‐
449	hsa_circ_0081039	.0218	2.5172	up	chr7	‐
450	hsa_circ_0051014	.0264	2.5148	up	chr19	100
451	hsa_circ_0040729	.0441	2.5125	up	chr16	100
452	hsa_circ_0030190	.0177	2.5062	up	chr13	28
453	hsa_circ_0026401	.0360	2.5058	up	chr12	10
454	hsa_circ_0132336	.0435	2.5026	up	chr6	4
455	hsa_circ_0058137	.0491	2.4929	up	chr2	21
456	hsa_circ_0054585	.0443	2.4891	up	chr2	1
457	hsa_circRNA3328‐4	.0418	2.4882	up	chr17	‐
458	hsa_circ_0049956	.0323	2.4881	up	chr19	2
459	hsa_circRNA11112‐4	.0485	2.4861	up	chr16	‐
460	hsa_circRNA5224	.0392	2.4771	up	chr22	15
461	hsa_circ_0051608	.0280	2.4755	up	chr19	6
462	hsa_circ_0034836	.0032	2.4749	up	chr15	100
463	hsa_circ_0069882	.0483	2.4733	up	chr4	3
464	hsa_circ_0061063	.0019	2.4665	up	chr20	25
465	hsa_circ_0076915	.0315	2.4563	up	chr6	‐
466	hsa_circ_0019676	.0392	2.4555	up	chr10	3
467	hsa_circRNA1461‐11	.0320	2.4550	up	chr11	6
468	hsa_circ_0080817	.0462	2.4404	up	chr7	32
469	hsa_circ_0128040	.0442	2.4404	up	chr5	8
470	hsa_circ_0074132	.0416	2.4372	up	chr5	4
471	hsa_circ_0019666	.0250	2.4312	up	chr10	5
472	hsa_circ_0110775	.0338	2.4309	up	chr1	84
473	hsa_circ_0020770	.0403	2.4298	up	chr11	81
474	hsa_circRNA783‐1	.0375	2.4056	up	chr1	1
475	hsa_circRNA13081‐7	.0365	2.4009	up	chr20	24
476	hsa_circ_0063732	.0380	2.3995	up	chr22	6
477	hsa_circ_0049471	.0372	2.3872	up	chr19	59
478	hsa_circ_0001553	.0389	2.3792	up	chr5	‐
479	hsa_circ_0139150	.0403	2.3791	up	chr9	4
480	hsa_circ_0046515	.0420	2.3777	up	chr17	5
481	hsa_circ_0000765	.0343	2.3772	up	chr17	‐
482	hsa_circRNA1883‐3	.0457	2.3743	up	chr12	2
483	hsa_circ_0003779	.0214	2.3703	up	chr10	9
484	hsa_circ_0046387	.0472	2.3686	up	chr17	98
485	hsa_circRNA693‐8	.0396	2.3671	up	chr1	1
486	hsa_circ_0119500	.0075	2.3637	up	chr2	2
487	hsa_circRNA499‐6	.0454	2.3560	up	chr1	4
488	hsa_circRNA11657‐4	.0220	2.3390	up	chr17	‐
489	hsa_circ_0085879	.0438	2.3288	up	chr8	7
490	hsa_circ_0020549	.0366	2.3279	up	chr10	2
491	hsa_circ_0004525	.0229	2.3249	up	chr20	4
492	hsa_circRNA4125‐2	.0221	2.3202	up	chr19	2
493	hsa_circ_0128024	.0302	2.3087	up	chr5	2
494	hsa_circ_0113803	.0304	2.3059	up	chr1	‐
495	hsa_circ_0019561	.0330	2.3036	up	chr10	3
496	hsa_circ_0048441	.0493	2.2988	up	chr19	69
497	hsa_circ_0104540	.0395	2.2893	up	chr15	14
498	hsa_circRNA11147‐16	.0288	2.2709	up	chr16	1
499	hsa_circ_0132953	.0432	2.2581	up	chr7	1
500	hsa_circ_0063099	.0444	2.2539	up	chr22	100
501	hsa_circ_0058314	.0439	2.2498	up	chr2	100
502	hsa_circRNA6675‐2	.0373	2.2444	up	chr6	3
503	hsa_circRNA2866‐179	.0226	2.2403	up	chr16	37
504	hsa_circ_0096997	.0426	2.2366	up	chr12	1
505	hsa_circ_0127068	.0343	2.2301	up	chr4	100
506	hsa_circ_0058139	.0303	2.2159	up	chr2	29
507	hsa_circ_0013816	.0420	2.1921	up	chr1	100
508	hsa_circ_0043736	.0472	2.1741	up	chr17	‐
509	hsa_circ_0079708	.0492	2.1714	up	chr7	20
510	hsa_circ_0028752	.0453	2.1518	up	chr12	100
511	hsa_circ_0076091	.0395	2.1488	up	chr6	‐
512	hsa_circRNA12187‐14	.0437	2.1445	up	chr19	3
513	hsa_circ_0058943	.0467	2.1413	up	chr2	100
514	hsa_circ_0098884	.0280	2.1348	up	chr12	1
515	hsa_circ_0037728	.0334	2.1331	up	chr16	19
516	hsa_circ_0081211	.0447	2.1331	up	chr7	16
517	hsa_circ_0026694	.0405	2.1295	up	chr12	4
518	hsa_circRNA13636‐3	.0026	2.1137	up	chr3	1
519	hsa_circRNA8528‐1	.0442	2.1105	up	chr1	1
520	hsa_circ_0056856	.0095	2.1067	up	chr2	2
521	hsa_circ_0001605	.0316	2.1065	up	chr6	44
522	hsa_circ_0098702	.0232	2.0930	up	chr12	28
523	hsa_circ_0065247	.0470	2.0878	up	chr3	40
524	hsa_circ_0018930	.0463	2.0803	up	chr10	100
525	hsa_circRNA5174‐30	.0383	2.0728	up	chr21	‐
526	hsa_circ_0032414	.0366	2.0701	up	chr14	49
527	hsa_circRNA7795‐18	.0485	2.0676	up	chr9	18
528	hsa_circ_0039734	.0499	2.0625	up	chr16	2
529	hsa_circRNA2382‐11	.0326	2.0545	up	chr14	55
530	hsa_circ_0038829	.0419	2.0523	up	chr16	2
531	hsa_circ_0094149	.0487	2.0263	up	chr10	‐
532	hsa_circ_0080476	.0008	2.0123	up	chr7	14
533	hsa_circ_0051355	.0473	2.0096	up	chr19	33
534	hsa_circ_0031019	.0278	2.0069	up	chr13	29
535	hsa_circ_0050576	.0162	2.0034	up	chr19	‐
						
1	hsa_circ_0066439	.0311	8.1982	down	chr3	100
2	hsa_circ_0054211	.0380	7.7496	down	chr2	‐
3	hsa_circ_0095920	.0204	7.7266	down	chr11	7
4	hsa_circ_0122984	.0215	7.0786	down	chr3	100
5	hsa_circ_0113067	.0389	6.9119	down	chr1	‐
6	hsa_circ_0039155	.0433	6.7674	down	chr16	9
7	hsa_circRNA4014‐45	.0295	6.4383	down	chr19	1
8	hsa_circ_0122979	.0200	6.1909	down	chr3	37
9	hsa_circ_0059665	.0208	5.8536	down	chr20	3
10	hsa_circ_0009319	.0119	4.4715	down	chr1	14
11	hsa_circ_0108269	.0466	4.0740	down	chr18	100
12	hsa_circRNA15520‐3	.0178	4.0661	down	chr8	4
13	hsa_circRNA7995‐20	.0323	3.8447	down	chrX	3
14	hsa_circ_0116473	.0198	3.5157	down	chr22	‐
15	hsa_circ_0072126	.0030	3.4456	down	chr5	3
16	hsa_circ_0138278	.0013	3.1260	down	chr9	24
17	hsa_circRNA4269‐5	.0028	2.9634	down	chr19	‐
18	hsa_circ_0024093	.0109	2.8327	down	chr11	100
19	hsa_circ_0072127	.0060	2.7938	down	chr5	‐
20	hsa_circRNA14838‐129	.0031	2.7874	down	chr6	100
21	hsa_circ_0048276	.0048	2.7553	down	chr19	11
22	hsa_circ_0087798	.0305	2.7281	down	chr9	100
23	hsa_circ_0072124	.0135	2.7164	down	chr5	43
24	hsa_circ_0051911	.0433	2.7030	down	chr19	2
25	hsa_circ_0015835	.0034	2.6614	down	chr1	13
26	hsa_circ_0083577	.0162	2.6553	down	chr8	100
27	hsa_circ_0109563	.0094	2.6242	down	chr19	8
28	hsa_circRNA9277‐12	.0033	2.5709	down	chr10	21
29	hsa_circ_0083773	.0054	2.5485	down	chr8	100
30	hsa_circ_0072695	.0109	2.5287	down	chr5	1
31	hsa_circ_0054909	.0109	2.5037	down	chr2	7
32	hsa_circ_0055714	.0487	2.4975	down	chr2	100
33	hsa_circ_0072118	.0178	2.4935	down	chr5	62
34	hsa_circ_0019871	.0011	2.4815	down	chr10	3
35	hsa_circ_0040298	.0024	2.4803	down	chr16	3
36	hsa_circ_0031515	.0001	2.4737	down	chr14	100
37	hsa_circRNA1322	.0425	2.4663	down	chr11	6
38	hsa_circ_0037397	.0003	2.4556	down	chr16	21
39	hsa_circ_0079644	.0205	2.4410	down	chr7	24
40	hsa_circ_0048377	.0057	2.4364	down	chr19	3
41	hsa_circ_0072696	.0073	2.4173	down	chr5	2
42	hsa_circ_0010232	.0021	2.3984	down	chr1	6
43	hsa_circ_0031872	.0023	2.3965	down	chr14	28
44	hsa_circRNA2454‐1	.0111	2.3875	down	chr14	1
45	hsa_circ_0098827	.0355	2.3841	down	chr12	6
46	hsa_circ_0021365	.0040	2.3776	down	chr11	100
47	hsa_circ_0129820	.0063	2.3762	down	chr5	1
48	hsa_circ_0077341	.0487	2.3622	down	chr6	2
49	hsa_circ_0043578	.0035	2.3564	down	chr17	2
50	hsa_circ_0052184	.0048	2.3548	down	chr19	‐
51	hsa_circRNA10194‐34	.0012	2.3485	down	chr12	1
52	hsa_circRNA3816‐16	.0002	2.3475	down	chr19	‐
53	hsa_circ_0073199	.0358	2.3331	down	chr5	9
54	hsa_circ_0077874	.0025	2.3325	down	chr6	‐
55	hsa_circ_0072702	.0246	2.3260	down	chr5	‐
56	hsa_circ_0079655	.0343	2.3179	down	chr7	‐
57	hsa_circ_0073196	.0172	2.3099	down	chr5	23
58	hsa_circ_0077873	.0190	2.2875	down	chr6	‐
59	hsa_circ_0073193	.0062	2.2837	down	chr5	23
60	hsa_circ_0077799	.0009	2.2833	down	chr6	‐
61	hsa_circ_0073640	.0453	2.2724	down	chr5	3
62	hsa_circ_0055072	.0035	2.2633	down	chr2	100
63	hsa_circ_0048764	.0032	2.2609	down	chr19	13
64	hsa_circ_0009272	.0021	2.2603	down	chr1	100
65	hsa_circ_0020796	.0029	2.2403	down	chr11	50
66	hsa_circ_0077317	.0312	2.2343	down	chr6	100
67	hsa_circRNA9277‐14	.0059	2.2323	down	chr10	1
68	hsa_circ_0043573	.0015	2.2293	down	chr17	16
69	hsa_circ_0041351	.0071	2.2214	down	chr17	‐
70	hsa_circRNA7333‐72	.0232	2.2150	down	chr7	1
71	hsa_circ_0050049	.0061	2.2079	down	chr19	3
72	hsa_circ_0106804	.0082	2.2010	down	chr17	100
73	hsa_circ_0023050	.0005	2.1980	down	chr11	67
74	hsa_circRNA14568‐1	.0050	2.1942	down	chr5	1
75	hsa_circ_0019876	.0204	2.1892	down	chr10	‐
76	hsa_circ_0001583	.0028	2.1863	down	chr6	‐
77	hsa_circ_0048576	.0092	2.1799	down	chr19	‐
78	hsa_circ_0118577	.0395	2.1789	down	chr2	100
79	hsa_circ_0015380	.0004	2.1688	down	chr1	47
80	hsa_circ_0049004	.0065	2.1611	down	chr19	14
81	hsa_circ_0085214	.0044	2.1552	down	chr8	100
82	hsa_circ_0050165	.0170	2.1461	down	chr19	100
83	hsa_circ_0018598	.0067	2.1300	down	chr10	27
84	hsa_circ_0076015	.0023	2.1297	down	chr6	100
85	hsa_circ_0050223	.0109	2.1242	down	chr19	44
86	hsa_circ_0083745	.0004	2.1192	down	chr8	58
87	hsa_circ_0018976	.0095	2.1189	down	chr10	100
88	hsa_circ_0030659	.0125	2.1185	down	chr13	3
89	hsa_circ_0086493	.0147	2.1158	down	chr9	61
90	hsa_circ_0068338	.0061	2.1138	down	chr3	4
91	hsa_circ_0107635	.0198	2.1038	down	chr17	‐
92	hsa_circ_0067970	.0063	2.0950	down	chr3	19
93	hsa_circ_0091117	.0123	2.0906	down	chrX	‐
94	hsa_circ_0028302	.0085	2.0879	down	chr12	50
95	hsa_circ_0050003	.0031	2.0856	down	chr19	100
96	hsa_circRNA3217‐22	.0065	2.0750	down	chr17	‐
97	hsa_circ_0000406	.0151	2.0714	down	chr12	1
98	hsa_circ_0062102	.0156	2.0675	down	chr21	66
99	hsa_circ_0009746	.0492	2.0614	down	chr1	6
100	hsa_circ_0124985	.0350	2.0607	down	chr4	‐
101	hsa_circ_0092229	.0027	2.0584	down	chrY	40
102	hsa_circ_0079649	.0182	2.0577	down	chr7	17
103	hsa_circ_0123224	.0135	2.0562	down	chr3	‐
104	hsa_circ_0086256	.0328	2.0482	down	chr9	14
105	hsa_circ_0042272	.0067	2.0476	down	chr17	6
106	hsa_circ_0069858	.0019	2.0442	down	chr4	100
107	hsa_circ_0075406	.0034	2.0400	down	chr5	1
108	hsa_circ_0077876	.0053	2.0356	down	chr6	‐
109	hsa_circ_0048545	.0082	2.0206	down	chr19	100
110	hsa_circ_0023381	.0348	2.0193	down	chr11	31
111	hsa_circ_0080145	.0128	2.0164	down	chr7	100
112	hsa_circ_0073886	.0005	2.0099	down	chr5	100
113	hsa_circ_0039941	.0110	2.0082	down	chr16	15
114	hsa_circRNA10930‐2	.0163	2.0068	down	chr16	1
115	hsa_circRNA4796‐2	.0298	2.0026	down	chr2	‐

### 3.3 Screening of target miRNAs for the differentially expressed circRNAs

**Figure 2:**
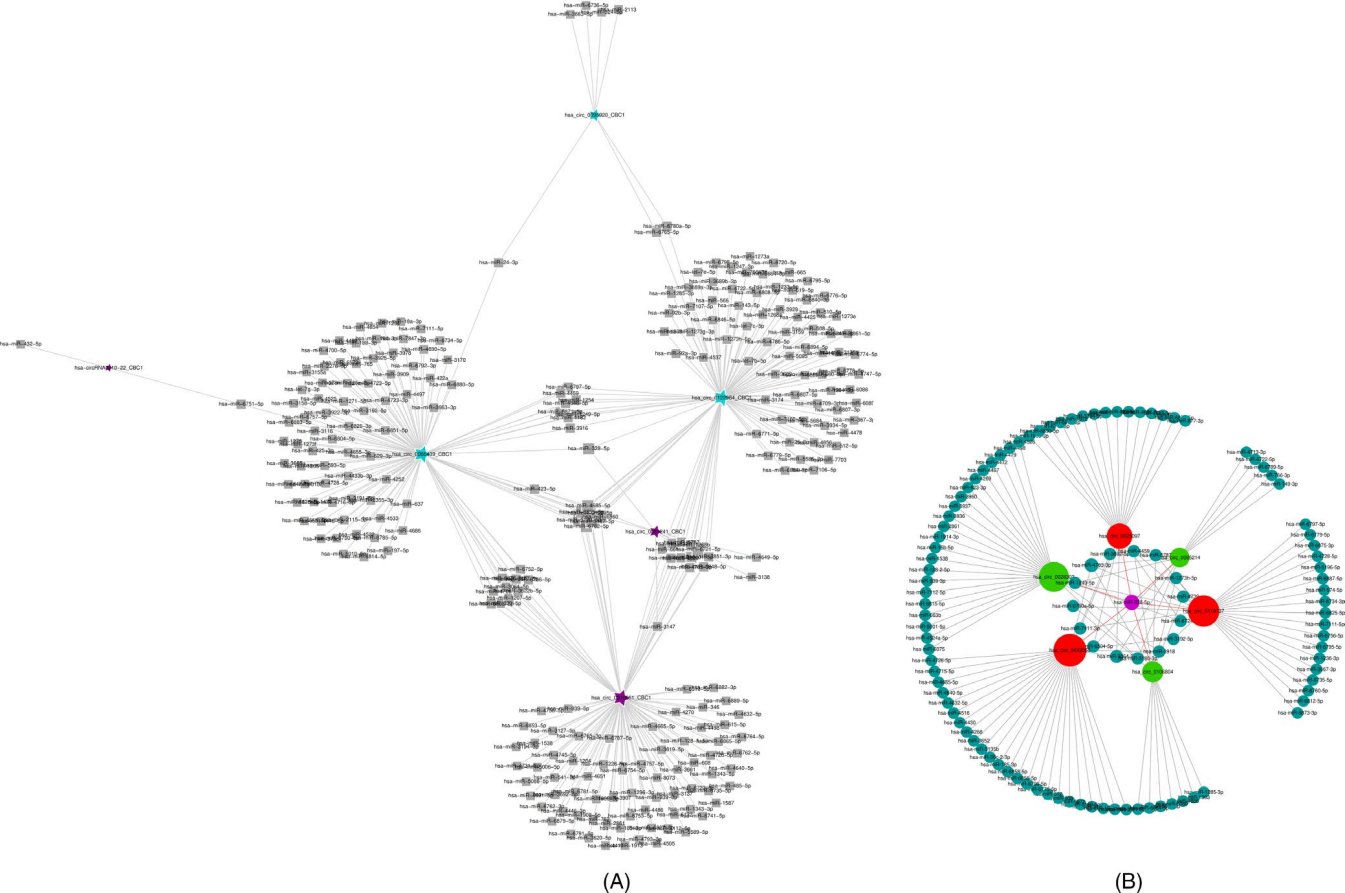
CircRNA‐miRNA network diagram. The larger the circRNA sphere, the greater the number of miRNAs that can be bound. (a) The top 10 differentially expressed circRNA corresponds to the circRNA‐miRNA network diagram. High expression level is indicated by purple and lower levels by blue. (b) The 6 circRNA candidates were able to bind to miR‐328‐5p. High expression level is indicated by red and lower levels by green

A total of 475 out of 650 circRNAs with different expressions could bind more than two miRNAs. Among the top 10 differentially expressed circRNAs, six had more than two target miRNAs, and their circRNA‐miRNA network relationship is shown in Figure 2A. The results show that there is a complex network relationship between circRNA and miRNA. Among them, RNA hsa_circ_0066439, hsa_circ_0081241, and hsa_circ_0122984 can regulate multiple signal pathways through miRNA hsa‐miR‐1254, hsa‐miR‐328‐5p, and other target miRNAs to participate in the AMI process. By further scrutinizing more stringent parameters, such as processed signal variation between repetitions and referencing to the established circRNA databases and publications, six new circRNA candidates were selected. The miRNA software was used to predict the target miRNAs of the six differentially expressed circRNAs. The results showed that the target miRNAs of the six differentially expressed circRNAs were larger than two. The circRNA‐miRNA network of these six circRNAs is shown in Figure 2B. It can be seen from this figure that RNA hsa_circ_0043563, hsa_circ_0119137, hsa_circ_0106804, and hsa_circ_0085214 can regulate multiple signal pathways through miRNA hsa‐miR‐4763‐3p, hsa‐miR‐328‐5p, etc Thus, it can participate in the occurrence of AMI. Subsequent hsa_circ_0025097 and hsa_circ_0028302 can also regulate multiple signal pathways through hsa‐miR‐328‐5p. Therefore, there are network relationships between circRNA‐miRNA that may be the key to their role in the pathogenesis and progression of AMI.

### 3.4 Enrichment analysis results for disease

**Figure 3:**
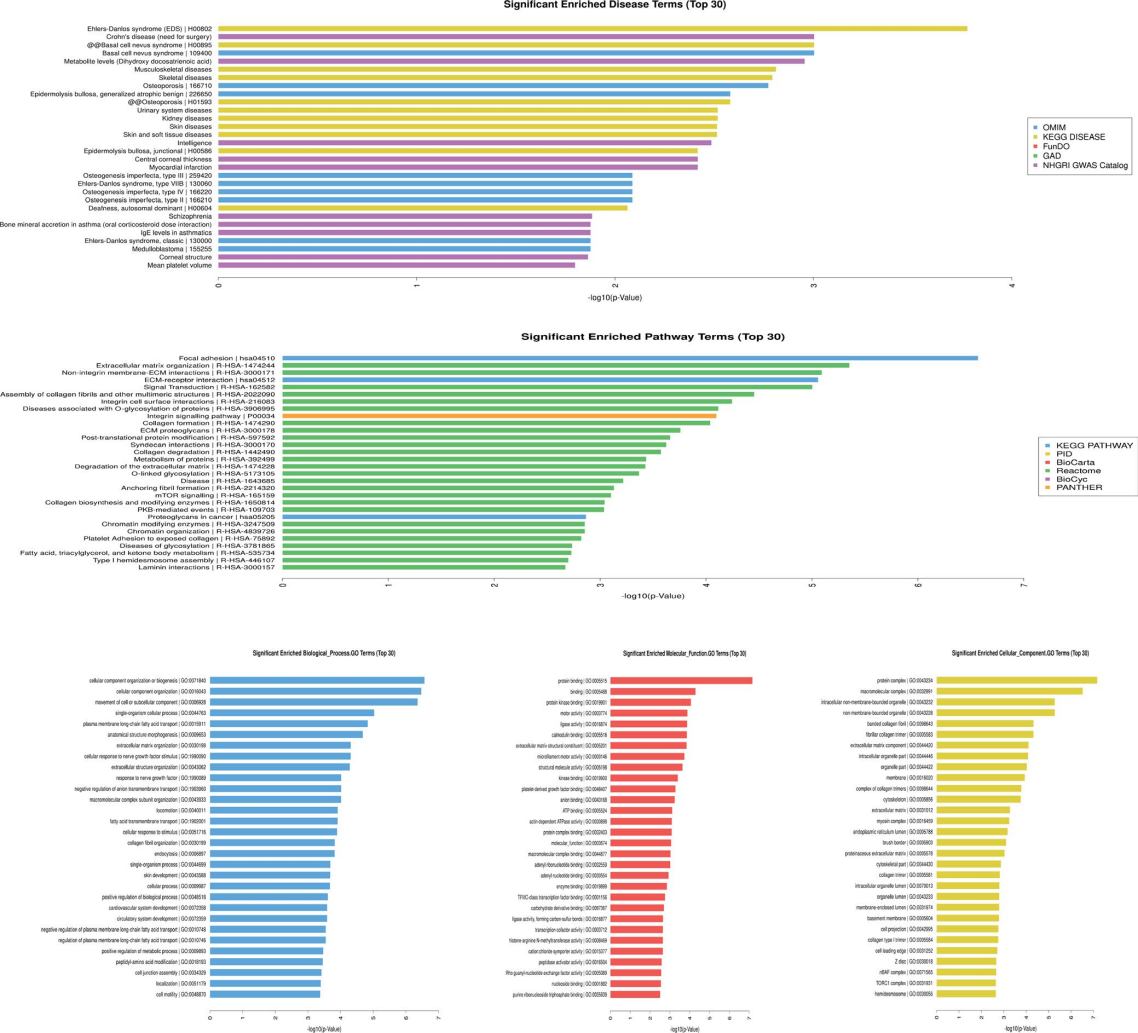
Top 30 significantly enriched disease, pathway, and GO analysis of circRNAs in AMI patients vs control group

Disease, pathway, and GO analysis suggest that these differentially expressed circRNAs are relevant to several vital biological processes, cellular components, molecular functions, and critical signaling pathways (Figure 3). By searching bioinformatics databases such as KEGG DISEASE, NHGRI GWAS Catalog, and OMIM, the significantly expressed genes in the whole blood samples of the patients with AMI were subject to enrichment analysis, and significance was marked by *P* < .05. The results are shown in Table 3. As can be seen from this table, after using 3 databases to perform a disease enrichment analysis of characteristic genes, 3 diseases closely related to the cardiovascular system were found, which were myocardial infarction, coronary heart disease, and hypertension. From the results, it can be determined that the target genes are closely related to coronary heart disease.

**Table 3:** Results from the disease enrichment analysis of 3 databases

Term	Database	*P*‐value
Myocardial infarction	NHGRI GWAS Catalog	.003822041
Coronary heart disease	NHGRI GWAS Catalog	.033700966
Hypertension	NHGRI GWAS Catalog	.015446158

### 3.5 Pathway analysis

To study the differentially expressed circRNAs in the whole blood of the patients with AMI, pathway enrichment analysis was performed using the databases of Reactome (http://www.reactome.org/), KEGG PATHWAY (http://www.genome.jp/kegg/), BioCyc (http://biocyc.org/), and PANTHER (http://www.pantherdb.org/). *P* < .05 was considered as a significant analysis, and the results are shown in Table 4.

**Table 4:** Results from the pathway enrichment analysis of 4 databases

Term	Database	*P*‐value
Chromatin organization	Reactome	.001389753
Chromatin‐modifying enzymes	Reactome	.001389753
Signal transduction	Reactome	.000009897
Lysine degradation	KEGG PATHWAY	.015878021
MAPK signaling pathway	KEGG PATHWAY	.022710463
Focal adhesion	KEGG PATHWAY	.000000269

Based on *P* < .05, no relevant pathways were found in the PANTHER or BioCyc databases, while in the KEGG PATHWAY database, it was suggested that lysine degradation, MAPK signaling pathway, and focal adhesion (*P* < .05) were involved in the pathogenesis of AMI and the process of disease development. The Reactome database prompted multiple pathways, such as signal transduction, chromatin organization, and chromatin‐modifying enzymes, as participating in the occurrence and development of AMI.

Based on the results of the pathway enrichment analysis performed using the various databases above, it is not difficult to discover that the target genes of these differentially expressed circRNAs of the AMI group can participate in the pathogenesis and disease development of AMI through some pathways. Many such pathways exist in the regulation of the development of AMI.

### 3.6 Gene ontology analysis

Similarly, gene ontology analysis was performed on the linear mRNA transcripts corresponding to 650 differentially expressed circRNAs selected from the specimens of the AMI group. The analysis included mainly molecular functions, biological processes, and cellular components.

**Figure 4:**
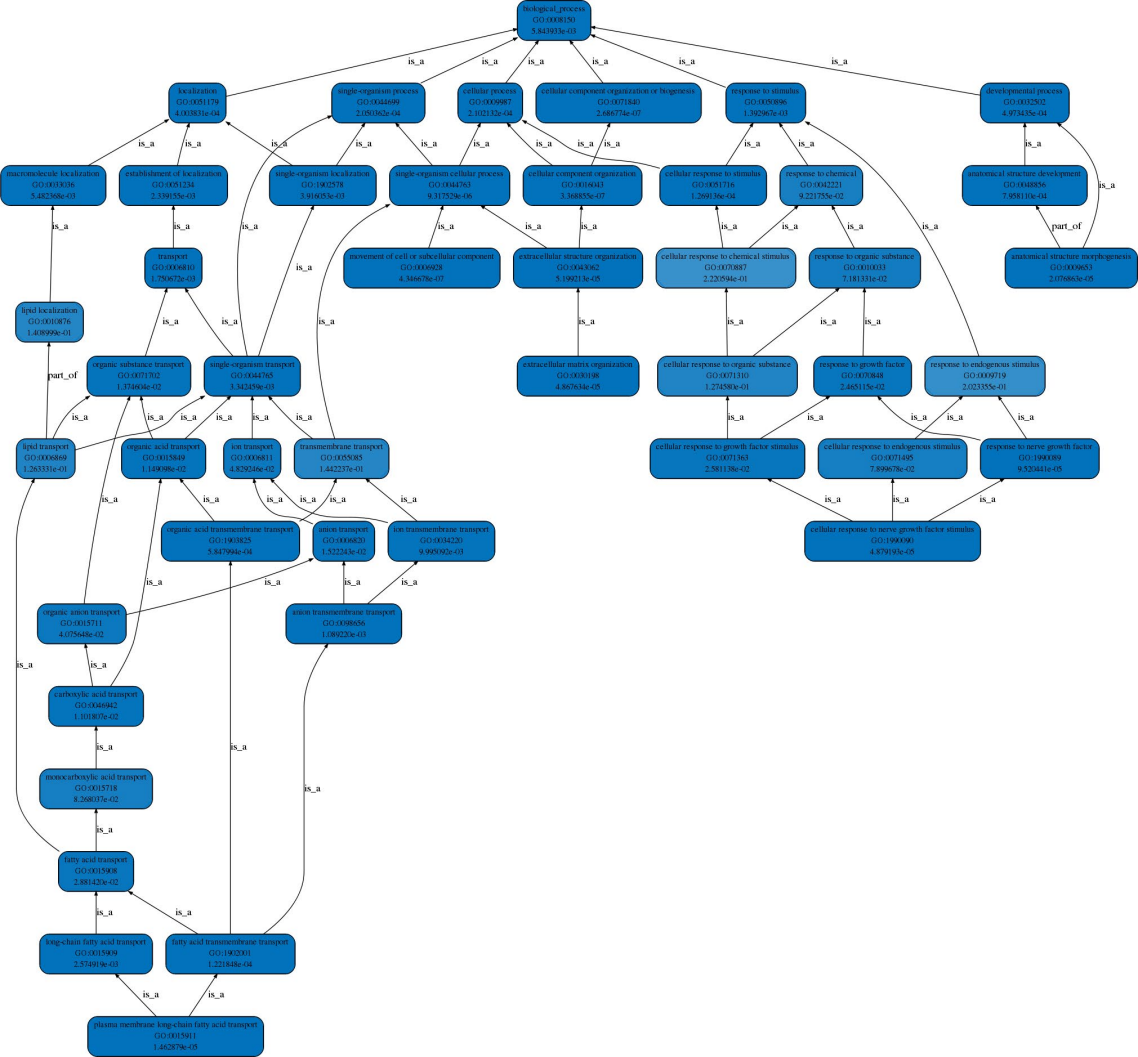
GO functional hierarchical network diagram (biological process). The first line of each node describes the GO node name, the second line describes the node number in GO, and the third line describes the corrected P‐value. The node color indicates the significance of the corrected P‐value, and the darker the color indicates the more significant the corrected P‐value

**Figure 5:**
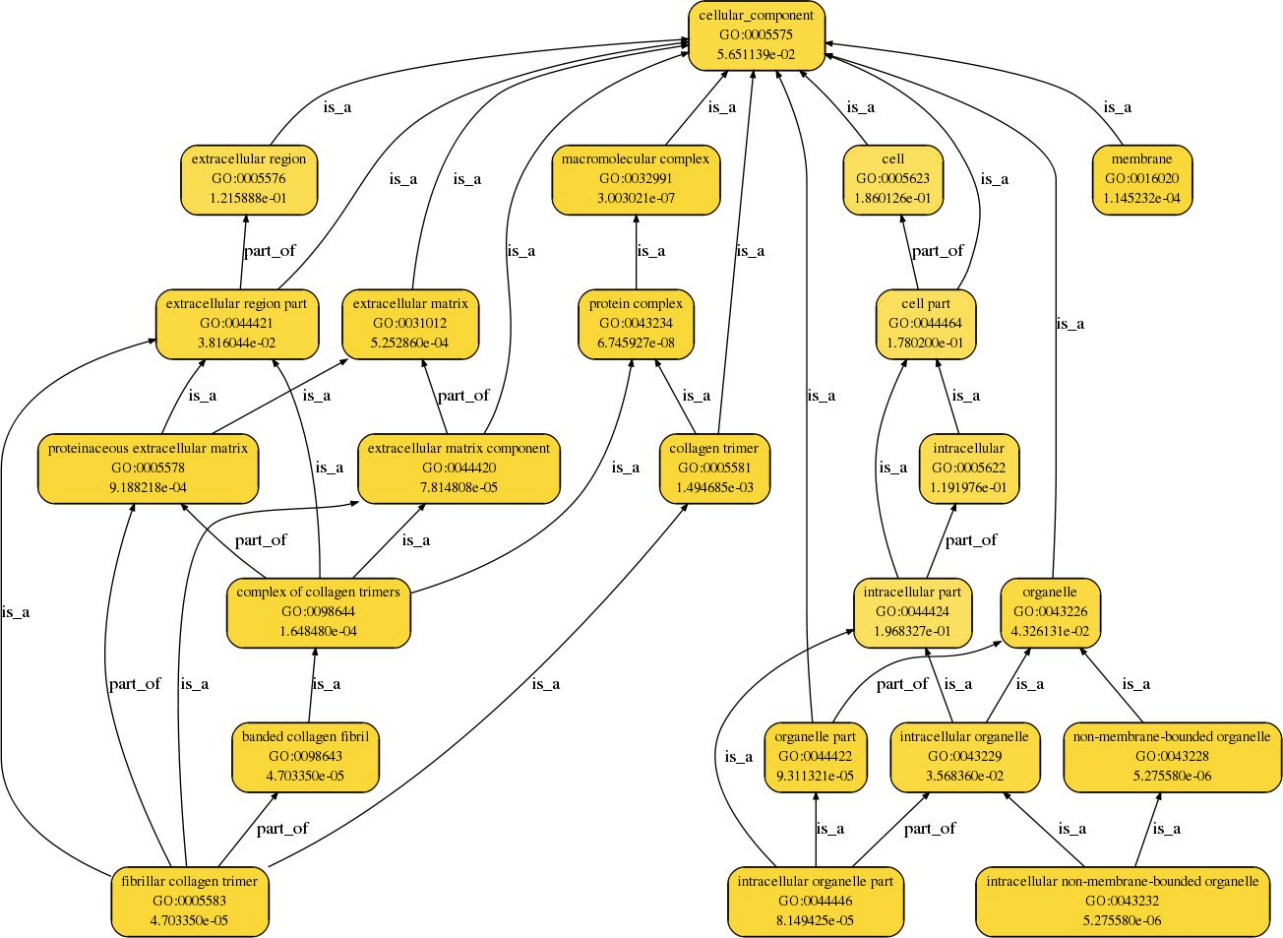
GO functional hierarchical network diagram (cellular component process). The first line of each node describes the GO node name, the second line describes the node number in GO, and the third line describes the corrected P‐value. The node color indicates the significance of the corrected P‐value, and the darker the color indicates, the more significant the corrected P‐value

**Figure 6:**
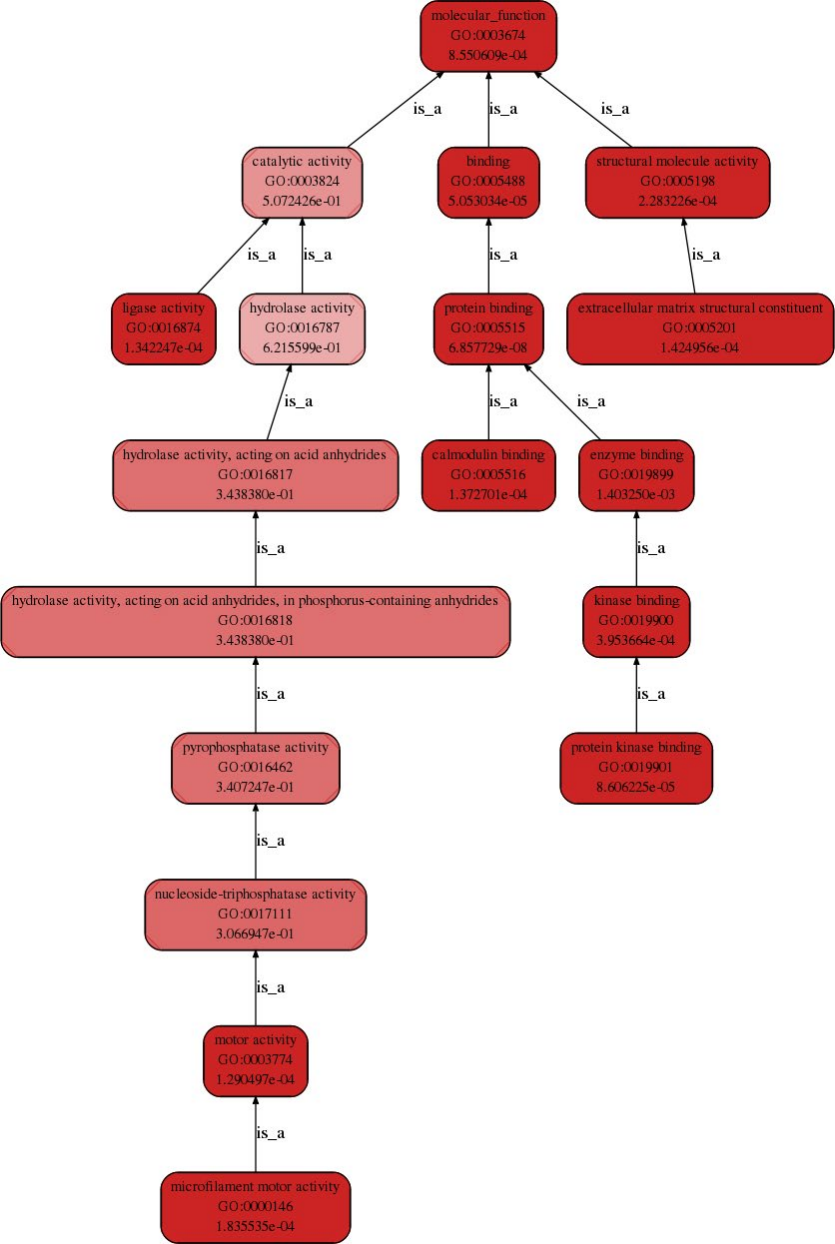
GO functional hierarchical network diagram (molecular function process). The first line of each node describes the GO node name, the second line describes the node number in GO, and the third line describes the corrected P‐value. The node color indicates the significance of the corrected P‐value, and the darker the color indicates the more significant the corrected P‐value

The gene ontology analysis demonstrated that the differentially expressed circRNAs play regulatory roles in cells through various biological processes, such as cellular, single‐organism, and cellular component organization (Figure 4); cellular components, such as protein complex, extracellular matrix, membrane, and extracellular matrix component (Figure 5); and molecular functions, such as binding and structural molecule activity (Figure 6).

## 4 DISCUSSION

Based on whether they can be translated, circRNAs were divided into noncoding circRNAs and coding circRNAs that have a closed circular structure and are not affected by exonucleases.^13^ Its expression is relatively more stable, and it is not easily degraded. In recent years, circRNA has emerged as a new member of the RNA family for that has attracted attention.^14^ In the past, studies of miRNAs made a series of advances to the understanding of the mechanisms in the actions of diseases, such as in heart development, cardiac hypertrophy, heart failure, arrhythmia, myocardial injury, apoptosis, and myocardial ischemia. In recent years, there have been reports in the literature that circRNA is related to CVDs. For example, Li et al have found that circRNA hsa_circ_0124644 can be used to diagnose coronary artery disease.^15^ Zhang et al discovered that circRNA MFACR inhibited the translation of MTP18 by competitively binding to miR‐652‐3p, thereby inhibiting mitochondrial division and affecting apoptosis, eventually playing a role in human heart disease.^16^ Furthermore, HRCR inhibits cardiac hypertrophy and heart failure, Cdrlas induces myocardial infarction, Circ‐Fox03 promotes heart aging, and cANRIL is associated with atherosclerosis.^17^ Because the circular structure of circRNA is more stable, it is easier to use as a potential new biomarker. miRNA is also a type of ncRNA, and it has been one of the most studied of these RNAs in recent years. It has been shown to play a very important role in the development of various diseases, and the role of miRNAs in CVDs has also gradually been discovered. Studies have found that circulating miRNA‐134, miRNA‐22, miRNA‐328, and miRNA‐499 have abnormal expression levels in the plasma of patients with AMI, suggesting that they may be potential biomarkers of AMI.^18^, ^19^, ^20^, ^21^ There are many miRNA response elements on circRNA. The sponge adsorption effect of circRNA on miRNA occurs mainly through response elements binding miRNA to block the inhibition of miRNA on its target gene expression. When circRNA is highly expressed, the target gene expression of miRNA is up‐regulated, and when circRNA is lowly expressed, the target gene expression of miRNA is down‐regulated.^22^ It can be said that the miRNA sponge function performed by circRNA plays an important role in the occurrence of diseases, competitively inhibiting the expression of miRNA and blocking the expression of miRNA on target genes. Therefore, the relationship between circRNA and AMI has great research potential. Since, as compared to linear structures, circRNA does not contain a poly A tail, it is not easily cleaved by exonuclease and exists more stably in an organism.^23^, ^24^ It also has high conservation, expression, and tissue specificity.^25^ Therefore, the regulation efficiency of circRNA is higher than that of linear structure RNA.^26^ These prior studies have indicated that circRNA is likely to have an important relationship with the occurrence of CVDs.

In this study, the circRNA expression in patients with AMI was analyzed using a microarray gene chip, and the circRNAs with significantly different expressions from controls were analyzed. The results showed that, as compared to the control group, 650 differentially expressed circRNAs were screened out for the AMI group, of which 535 were up‐regulated and 115 were down‐regulated (Table 2, Figure 1C). Such a large number of circRNA differential expression results indicate that circRNA is likely to play a positive role in the occurrence and development of AMI. The circRNA‐miRNA network was constructed after screening out the differentially expressed circRNAs, and it showed that 475 differentially expressed circRNAs could bind > 2 miRNAs. Among the 10 circRNAs with the largest difference, three of them can bind to miR‐328‐5p‐related cardiovascular diseases, including one up‐regulated hsa_circ_0081241 and two down‐regulated hsa_circ_0066439 and hsa_circ_0122984 (Figure 2A). In addition, the 6 novel circRNA candidates were able to bind to miR‐328‐5p related to myocardial infarction (Figure 2B), including 3 up‐regulated (hsa_circ_0043563, hsa_circ_0119137, and hsa_circ_0025097) and 3 down‐regulated (hsa_circ_0106804, hsa_circ_0085214, and hsa_circ_0028302). In a previous study, He et al^18^ found that miR‐328 and miR‐134 have important diagnostic value for AMI, and levels of miR‐328 and miR‐134 are related to mortality or an increased risk of developing heart failure. Wang et al^19^ reported that levels of miR‐328 in the plasma and whole blood of AMI patients were significantly increased: 10.9 times and 16.1 times as compared to a control group, respectively. The level of miR‐133 increased 4.4 times, suggesting that miR‐328 and miR‐133 may represent novel biomarkers of AMI.

Among the previous studies, Ruan et al^27^ have explored the possible regulatory mechanisms of circRNA‐miRNA regulatory networks in the pathogenesis and progression of gastric cancer. They discovered the circRNA‐miRNA‐mRNA pathway and indicated that a possible role for circRNA in the occurrence and development of gastric cancer may be helpful to developing potential tools for the early diagnosis and effective treatment of gastric cancer. It should be noted that the circRNA‐miRNA‐mRNA axis has become the most studied mechanism for the occurrence and development of gastric cancer. Dudekula et al^28^ have constructed a new database for searching the open databases of circRNAs, miRNAs, and RNA‐binding proteins to help provide bioinformatics analyses of the binding sites on circRNA. By using this database, we can also search for the binding sites of related circRNA and build a general regulatory network. Recent studies have also shown that identifying the circRNA‐miRNA network provides new insights into the prognosis and treatment of breast cancer.^29^ In addition, some scholars have proposed that the regulatory network of circRNA‐miRNA‐mRNA also exists in plants and have pointed out that this network is likely to affect the development of flowers; this pathway may also play a potential regulatory role in the response of plants to external environmental stress.^28^ The results of these many studies fully show that the circRNA‐miRNA‐mRNA network may be ubiquitous in the occurrence and development of various diseases. This pathway and network can regulate the occurrence and progression of various diseases and disease outcomes. The results of the current study have shown that multiple circRNAs can bind to miR‐328‐5p, which is associated with myocardial infarction. The possibility of a network correlation between the circRNA and miRNA was first confirmed, and then, it was suggested that this network correlation pathway is likely to have a certain regulatory mechanism during the occurrence and progression of AMI. Thus, this regulatory mechanism will affect the occurrence, development, and prognosis of AMI. This is of great significance for identifying circRNA as a possible biomarker for AMI and revealing the complex regulatory mechanisms in the process of AMI. According to the results of enrichment analysis and gene ontology analysis, it can be found that the circRNA‐miRNA pathway is likely to exist in the occurrence and development of AMI, such as lysine degradation, MAPK signaling pathway, and focal adhesion (Table 4). Combined with the screened circRNAs and bound miRNAs, this pathway is likely to participate in the process of regulating the occurrence and development of AMI.

There are some limitations in this study. First, the sample size was small, and the sample was only obtained from six subjects. Second, there is no verification of RT‐PCR for the top circRNA candidates expressing differences. In the future, circRNAs, such as hsa_circ_0066439, hsa_circ_0043563, hsa_circ_0119137, and miR‐328‐5p, should be verified to analyze the levels and interactions of these new circRNAs with miR‐328‐5p.

## 5 CONCLUSION

There were 650 circRNAs that were differentially expressed in AMI disease, and an interaction between circRNA and miRNA is involved in the occurrence and development of AMI. By combining the results of the disease enrichment analysis, pathway enrichment analysis, and gene ontology analysis, it can be found that the circRNA‐miRNA interaction pathway very likely participates in regulating the occurrence and development of AMI.

## 6 COMPETING INTERESTS

The authors declare that they have no competing interests.

## AUTHOR CONTRIBUTIONS

YH Tang involved in protocol/project development and final approval of the version to be submitted. MH Jiang collected or managed the data. LL Yin analyzed the data, and wrote the study.

## Data Availability

The data used to support the findings of this study are available from the corresponding author upon request.
